# Investigating the Potential of Transparent Parallel-Arranged Micro-Perforated Panels (MPPs) as Sound Absorbers in Classrooms

**DOI:** 10.3390/ijerph20021445

**Published:** 2023-01-13

**Authors:** Ela Fasllija, Semiha Yilmazer

**Affiliations:** Department of Interior Architecture and Environmental Design, Faculty of Art, Design and Architecture, Bilkent University, Ankara 06800, Turkey

**Keywords:** acoustic comfort, reverberation time, speech intelligibility, sound-absorbing materials, resonators, micro-perforated panels

## Abstract

Acoustic deficiencies due to lack of absorption in indoor spaces may sometime render significant buildings unfit for their purpose, especially the ones used as speech auditoria. This study investigates the potential of designing wideband acoustic absorbers composed of parallel-arranged micro-perforated panels (MPPs), known as efficient absorbers that do not need any other fibrous/porous material to have a high absorptive performance. It aims to integrate architectural trends such as transparency and the use of raw materials with acoustical constraints to ensure optimal indoor acoustic conditions. It proposes a structure composed of four parallel-arranged MPPs, which have been theoretically modelled using the electrical Equivalent Circuit Model (ECM) and implemented on an acrylic prototype using recent techniques such as CNC machining tools. The resulting samples are experimentally analysed for their absorption efficiency through the ISO-10534-2 method in an impedance tube. The results show that the prediction model and the experimental data are in good agreement. Afterward, the investigation focuses on applying the most absorptive MPP structure in a classroom without acoustic treatment through numerical simulations in ODEON 16 Acoustics Software. When the proposed material is installed as a wall panel, the results show an improvement toward optimum values in Reverberation Time (RT30) and Speech Transmission Index (STI).

## 1. Introduction

A person perceives the acoustic quality of space on three different levels: aesthetically, relating to the architecture; ergonomically, relating to the spatial layout, and functionally, relating to how well the sound waves are transmitted into space [1]. Acoustic comfort is defined as the perceived state of well-being and satisfaction with the acoustic conditions in an environment [2]. The ultimate goal of achieving acoustic comfort, which is both a qualitative and quantitative procedure, is the reduction in possible variables that can provoke discomfort in occupants. For instance, to reduce noise levels from outdoors and indoors, a quantitative evaluation of parameters, such as sound insulation and sound absorption coefficients of building and finishing materials, is considered [3].

When sound enters a space or originates in that space, its behaviour is affected by the room materials and its volume. The hardness and the stiffness of bare materials used as finishes (stone, metal, glass, and plaster) in the built environment cause the repeated reflectance of the sound waves, resulting in auditory problems such as high reverberation and amplified noise, especially in schools and offices [4]. Issues related to acoustic comfort seem to be the rule rather than the exception in indoor education environments, making them unfit for their purpose [5,6,7,8,9].

Literature has highlighted the positive association of good acoustic conditions in classrooms with higher well-being, acoustical satisfaction, and concentration [10,11]. In contrast, external and internal noise seem to significantly impact the performance in math, science, and literacy of primary school children [12], their cognitive skills [13], and their arousal and attention [14]. Knowing that long reverberation time amplifies the existing ambient noise, the chronic effects of long reverberation have negatively affected users’ well-being in educational environments [15].

Based on studies that thoroughly assessed educational facilities’ acoustic conditions, benchmarks for room acoustics parameters (reverberation time, speech intelligibility, and background sound levels) for classrooms have been established in current standards and regulations [16,17,18,19,20]. For speech purposes, it is equally crucial to ensure good speech intelligibility for the audience [21] and prevent the excessive vocal effort of the speaker [22]. Speech intelligibility, defined as the percentage of correctly understood speech items compared to the overall, is directly affected by Reverberation Time (RT) and speech signal-to-noise ratio (SNR) [23]. It is empirically measured with acoustic parameters such as the Speech Transmission Index (STI) and Clarity (C50). Various acoustic treatments, such as absorbers and diffusers, can control RT. The former are adequate for reducing the reverberant sound level, while the latter permit uniformly distributed sound energy in the room [24].

Absorbers are commonly used to improve acoustic comfort by reducing reverberation and sound levels in closed spaces. For Schmitz [25], acoustic treatments in classrooms combine porous and resonant absorbers that absorb in “nearly all relevant frequency ranges.” A state-of-the-art on absorbing materials shows that passive absorbers such as fibrous or porous materials are efficient for damping high-frequency noises [26], but problems are increasingly found at low frequencies [1]. Conventional dissipative materials (mineral wool, fiberglass, acoustic plaster and gypsum, foam, etc.) can absorb the sound energy from waves whose wavelengths are no greater than about four to eight times the thickness of their layer thickness. Even though mineral fibres have proven to be effective in terms of thermal insulation and high-frequency acoustic attenuation, they have a fragile and open structure that can cause severe soiling and hygiene problems. Depending on the type of exposure, they can cause respiratory issues and inflame the eyes and skin [27,28]. Furthermore, given the high energy demand for these mineral fibres during the manufacturing process and the difficulties in safely disposing them at the end of their working life, alternative absorbing materials are being widely researched [29]. One trend of studies is concerned with investigating the potential of available natural fibres [28,30], while other approaches are focused on engineered materials [26] that are set forth due to the advances in technology and manufacturing processes.

For treating low-frequency problems, resonant structures are usually used. These reactive structures are mass–spring systems with damping to absorb the system’s resonant frequency. They are most commonly used in room acoustics in the form of membranes, Helmholtz resonators, and perforated, slotted, and micro-perforated panels (MPPs) [24]. The latter are alternative fibreless absorbers that can cope with the highest hygiene demands but usually only offer narrowband absorption. In the 1970s, Maa [31] found that if the cross-sectional scale of the perforated holes is in the submillimetre range, losses will occur due to viscous boundary effects in the perforations. Their acoustic effectiveness can be precisely adjusted almost independently of the material selection but solely by choice of their geometric parameters (*d*-hole diameter, *t*-panel thickness, *p*-perforation ratio, and *D*-length of cavity behind the panel). Since the absorption performance principles are independent of the material’s type, MPPs can be integrated into diverse materials such as plastics, glass, wood, and metal [32]. They can be made of transparent or colourful plates or membranes, making them in demand from architects for sound quality control in the built environment [1].

Due to the handicap of having a narrow absorption bandwidth, several structures based on MPP arrangements in series and parallel have been explored to broaden the absorption bandwidth by introducing multiple resonances. Maa first proposed a double-layer MPP absorber [33], based on which several studies involving multiple-layer MPPs have followed [34,35,36]. Sakagami and colleagues [37] explored two parallel-arranged MPP resonators, including associated panels and cavities, with or without interior partitions, yielding good agreement between theory and measurement. The authors of [38,39,40] all studied the different potential parallel-arranged MPPs on the system’s absorption coefficient. These studies show that the absorption bandwidth is expanded to lower frequencies due to the additional multi-resonance peaks. Lately, different authors have been investigating other possibilities of the design cavity behind the MPPs, which can be coiled [41], L-shaped [42], J-shaped [43], or Archimedean-inspired [44]. Those innovations have set the ground for the realization of complex structures that exhibit acoustic properties not found in nature [26]. Moreover, the hybrid serial–parallel MPP structures designed on polymers [45,46,47,48,49,50] have shown that the correct combination of multiple parameters may achieve a more efficient and wideband sound absorption.

The literature shows that the models continue in the field of engineering. However, there is not enough information about the transition of these models to architectural applications and their performance. From an acoustic point of view, achieving a wideband absorption, designing for a maximum functional area, and preserving the flexibility of the interiors may not be possible while using permanently mounted thick absorbers such as conventional porous and fibrous materials. An alternate remedy is eliminating the destructive reflections by employing selective absorption or absorption by design [1]. Beyond designing for appropriate reverberation time and speech intelligibility in classrooms, architects ought to make sensible choices regarding the finishing materials. They need to avoid health-harming, toxic components and embrace materials promoting physical, mental, and environmental health.

In this light, this study is focused on room acoustics with a general expectation of better acoustic performance, better visual aesthetics, and healthy materials. Its main goal is to investigate different arrangements of transparent MPP absorbers using the Equivalent Circuit Model (ECM) to achieve a wider absorption bandwidth. The paper begins by initially computing and then experimentally testing the sound absorption behaviour of two different MPP arrangements. Then, after manufacturing their respective samples using digital manufacturing tools, their absorption coefficients are measured using the transfer function method in the impedance tube, according to ISO-10534-2 [51]. As a consequent and second goal, this study aims to transition to architectural spaces by understanding how the previously suggested absorbers affect the room acoustics parameters (RT and STI) of a classroom that has not been treated in terms of acoustics. The status quo of the acoustic conditions of the case study is measured in situ and calibrated in geometrical acoustics simulations. Lastly, the same acoustic assessment is conducted after the best-performing MPP absorber has been introduced as a wall panel in the classroom.

## 2. Materials and Methods

### 2.1. Theoretical Background

#### 2.1.1. Acoustic Impedance of MPPs According to ECM

**Figure 1:**
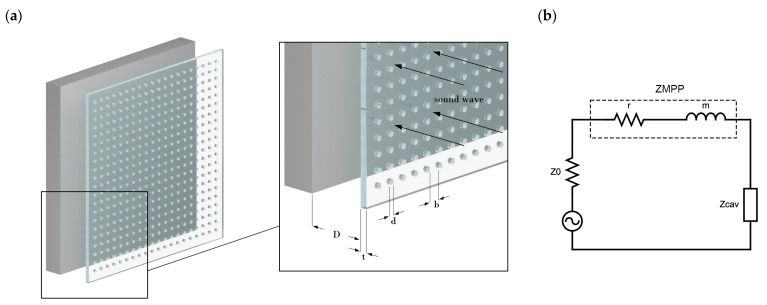
(**a**) An MPP and its geometrical parameter and (**b**) its equivalent circuit.

The basic system of a single-layer MPP (Figure 1a) consists of a micro-perforated panel, a rigid back wall, and the air cavity in between, where *d* is the hole diameter, *t* is panel thickness, *b* is the distance between the holes, constituting *p* as perforation ratio, and *D* is the distance from the back wall. When the dimensions of those regions are much smaller than the wave of interest, then an electro-acoustic analogy can be implemented to derive an equivalent circuit [31], as seen in Figure 1b.

The acoustic impedance of the MPP ($Z_{MPP}$) in Equation (1) is composed of a real part denoted as *r* (Equation (2)), constituting the resistive part of the panel, more specifically, the viscous losses at the edges. On the other hand, *m* (Equation (3)) is the imaginary part, corresponding to the reactance of the inertial motion of air in the small holes.

(1) $$Z_{MPP}=Z_{resistance}+Z_{reactance}=r+j\omega m$$

(2) $$r=\frac{32\eta t}{d^{2}p}\left[\sqrt{1+\frac{x^{2}}{32}}+\frac{\sqrt{2}x}{8}\frac{d}{t}\right]$$

(3) $$m=\frac{\rho t}{p}\left[1+\frac{1}{\sqrt{9+\frac{x^{2}}{2}}}+0.85\frac{d}{t}\right]$$

Here, x=d2ωρη is the perforation constant, *ω* = 2*πf* is the angular frequency, η is viscosity (1.6 × 10^−5^ m^2^/s), and *ρ* = air density (1.204 kg/m^3^), with *d*, *p*, and *t* being the hole diameter, perforation ratio, and thickness of the panel, respectively. When the MPP is placed at a distance from the wall, the resulting surface impedance ($Z_{tot}$) (Equation (5)) adds the impedance of the panel with the impedance of the air cavity ($Z_{cav}$), having a depth of *D* (Equation (4)).

(4) $$Z_{cav}=-j\rho c\cot\left(\frac{\omega D}{c}\right)$$

(5) $$Z_{tot}=Z_{MPP}+Z_{cav}$$

The sound absorption coefficient for a normal incidence sound wave can be calculated as follows, where ρc is the characteristic impedance of air.

(6) $$\alpha =1-{\left|\frac{Z_{tot}-\rho c}{Z_{tot}+\rho c}\right|}^{2}$$

#### 2.1.2. Parallel-Arranged MPPs (Prototype 1)

**Figure 2:**
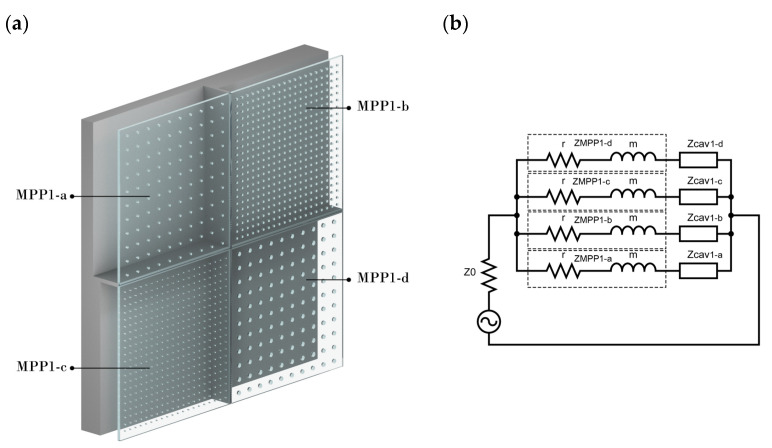
(**a**) A schematic diagram of four parallel-arranged MPPs with partitioned cavities and (**b**) its respective equivalent circuit.

Using the electric Equivalent Circuit Model (ECM), different MPP combinations in series and/or parallel can be designed to broaden the absorption spectrum band. When *n* number of MPPs are connected in parallel, each having a specific cavity partitioned from another as seen in Figure 2a, the total impedance of the panel is calculated as follows:

(7) $$Z_{a}=Z_{MPP1a}+Z_{Cav1a}$$

(8) $$Z_{b}=Z_{MPP1b}+Z_{Cav1b}$$

(9) $$Z_{c}=Z_{MPP1c}+Z_{Cav1c}$$

(10) $$Z_{d}=Z_{MPP1d}+Z_{Cav1d}$$

Thus:

(11) $$Z_{tot}={\left(\frac{\varphi_{a}}{Z_{a}}+\frac{\varphi_{b}}{Z_{b}}+\frac{\varphi_{c}}{Z_{c}}+\frac{\varphi_{d}}{Z_{d}}\right)}^{-1}$$

Here, $Z_{MPP1i}$ is the total acoustic impedance of each MPP, $Z_{Cav\left(i\right)}$ denotes each respective cavity after a specific MPP, and *φ* is the ratio each panel has to the overall surface panel. An example of this combination with its equivalent circuit is shown in Figure 2b.

In the scope of this study, a combination of parameters, as shown in Table 1, has first been numerically investigated. The authors of [52] show that the accepted range for MPP hole diameters is from 0.1–2 mm, and the accepted range for panel thickness is from 0.2–30 mm. The values chosen for this study are based on intuitive theoretical predictions that are concerned at the same time with manufacturing limitations. The material was assumed to be rigid so that it does not vibrate and has enough mechanical strength when used as a wall/ceiling panel. Each subpanel of MPP1 (MPP1-a, MPP1-b, MPP1-c, MPP1-d) has different perforations and back cavities partitioned, so that each of them faces a specific cavity (*D*) tuned at a different resonant frequency.

#### 2.1.3. Double-Layer Parallel-Arranged MPPs (Prototype 2)

**Figure 3:**
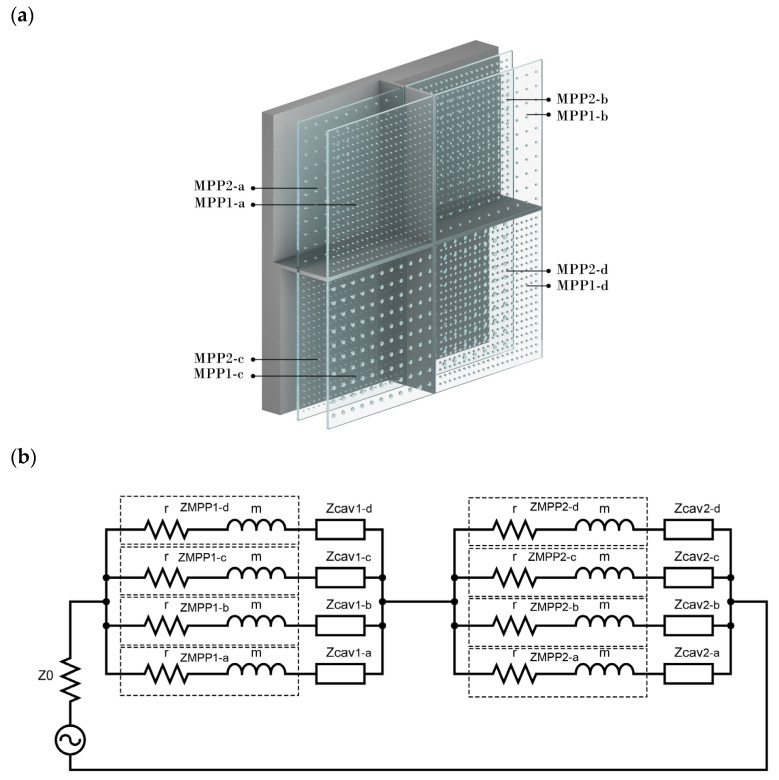
(**a**) Two layers of parallel-arranged MPPs with different back cavities. (**b**) The respective equivalent circuits of two layers of parallel-arranged MPPs with different back cavities after each layer.

Moreover, a wider band absorption can also be achieved by cascading multiple MPP layers into a composite [34]. The measured acoustic impedance shows the relation between the initial one, the change it undergoes in a transmission region, and the outgoing pressure and particle velocity. To gain a broader insight into the absorption coefficient of such structures, this study investigated two layers (MPP1 and MPP2), each having four parallel-arranged MPPs (MPP1-a, MPP1-b, MPP1-c, and MPP1-d and MPP2-a, MPP2-b, MPP2-c, and MPP2-d, respectively). Their parameters are listed in Table 2. The back cavity after MPP2, which is the layer facing the rigid wall, has four isolated sub-cavities, each (*D*2) ranging from 20 to 80 mm. Furthermore, the cavities after the first MPP1, the layer which welcomes the sound wave, are partitioned into four, even though it (*D*1) has the same length of 20 mm. Figure 3a is a graphical demonstration of such a system.

The ECM arrangement shown in Figure 3b shows that each acoustic impedance of MPP1 and MPP2 can be calculated by using Equation (7). The impedance of each cavity (*D*1, *D*2) facing each MPP layer can be predicted by using Equation (3). Moreover, the surface impedance is calculated by the following equation (Equation (12)) based on ECM and results from [47], where $Z_{tot\left(i\right)}$ is the overall specific acoustic impedance of each subpanel of MPP1. The absorption coefficient can be therefore calculated by Equation (6).

(12) $$Z_{tot\left(i\right)}=Z_{MPP1i}+{\left(\frac{1}{Z_{cav\left(D1\right)}}+\frac{1}{Z_{MPP2i}+Z_{cav\;\left(D2\right)}}\right)}^{-1}$$

### 2.2. Experimental Setup

This section includes the production of the prototypes theoretically modelled in the previous section. It goes on to validate the samples’ absorption coefficient through experiments in an impedance tube.

#### 2.2.1. Production of Samples

**Figure 4:**
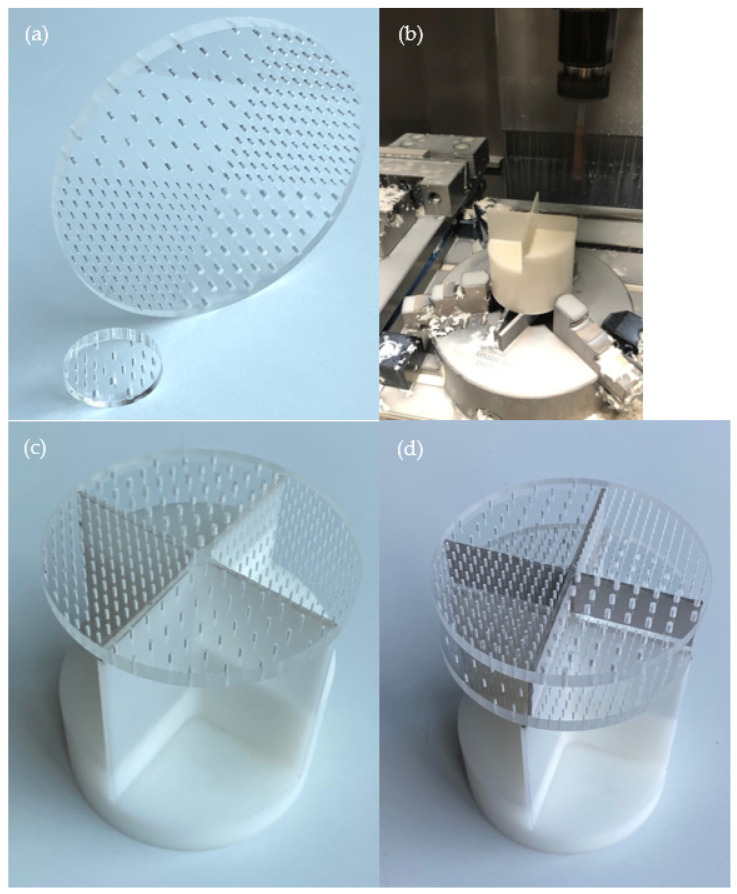
(**a**) Acrylic prototypes; (**b**) CNC machining tools etching the back cavity structure; (**c**) Prototype 1—One-layer parallel-arranged MPPs; (**d**) Prototype 2—Double-layer parallel-arranged MPPs.

The production of previously explained MPP combinations was finalized on 6 mm thick acrylic glass panels (density = 1190 kg/m^3^), a commonly used material when transparency in interior spaces is requested. The MPP circular prototypes with diameters of 100 mm, seen in Figure 4a, were designed and sent to the fabrication tools such as CNC and laser cutter at National Nanotechnology Research Centre (UNAM), Bilkent University. CNC stands for Computerized Numerical Control and is a computerized manufacturing process in which pre-programmed software and code control the movement of production equipment [53]. Different nozzle tips (from 0.5 mm to 2 mm) were used to open the micro-perforations by applying different cutting speeds and pressures until the desired neatness and precision were achieved. The back part was produced from an ABS (Acrylonitrile Butadiene Styrene) cylindrical mould (See Figure 4b), a thermoplastic material widely used due to its relatively low cost and excellent toughness. Prototypes with nearly 100 mm and 30 mm diameters were manufactured based on normal incidence absorption data according to TS EN ISO 10354-2 [51]. The parallel-arranged MPP layers were attached to the partitions with double-sided tape to form the final structure (Figure 4c,d). The structure was fit as a whole, and since it did not have any other enveloping structure, the walls of the impedance tube were supposed to form the specific back cavities for each MPP. The overall thickness for Prototype 1 was 86 mm, and it was 112 mm for Prototype 2.

#### 2.2.2. Assessment of Absorption Coefficient

**Figure 5:**
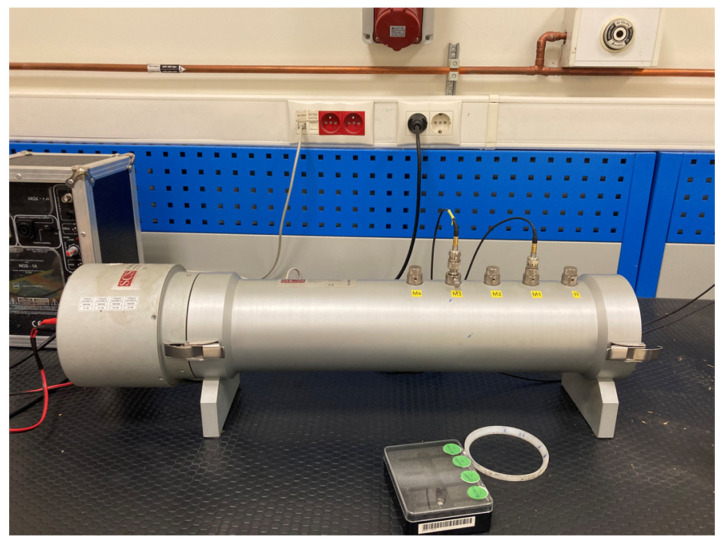
Kundt’s impedance tube at TSE (Tuzla, Istanbul).

Experimental validations were conducted for the two different scenarios of the prepared acrylic prototypes (Prototype 1—one-layer parallel-arranged MPPs; Prototype 2—Double-layer parallel-arranged MPPs). Due to feasibility issues and as many previous studies have done [37,40,54], the samples’ absorption coefficient was measured utilizing a two-microphone impedance tube mechanism following the standard TS EN ISO 10354-2 [51] at the Turkish Standard Institution (TSE), an accredited building materials acoustic laboratory located in Tuzla, Istanbul (Figure 5).

Before the measurements, the speaker was run for ten minutes to stabilize the temperature inside the tube. The environment’s temperature was 22° Celsius, and the relative humidity was 37%. The sample was mounted and sealed with special tape inside the tube so that it did not protrude from the front face of the sample holder and did not leak air around it. Sound absorption coefficient measurements were performed in the frequency ranges of 100–1600 Hz (100 mm sample) and 1600–6300 Hz (30 mm sample) in 1/3 octave bands as the standard requires. The SCS 9020B Impedance Kundt tube’s system works as follows: a sound pressure wave, generated by a signal generator with the help of a speaker, is sent into the tube. This wave, by hitting the fixed sample at the other end of the tube, absorbs some of its energy and comes back with reduced amplitude. The transfer function is obtained by measuring the sound pressures generated by two microphones having a distance of 100 mm, and therefore, sound absorption coefficient is calculated. The advantage of this method is that surface impedance and sound absorption coefficient values are achieved in a single measurement; however, it gives insights only for plane waves. To test the reproducibility of the materials, each measurement was repeated with three samples. 

### 2.3. Case Study

The final part of the study aims to assess the performance of the proposed prototypes in a real room acoustic scenario. To address this problem, a graduate studio classroom without any acoustic treatments in the Department of Interior Architecture and Environmental Design of Bilkent University was selected.

#### 2.3.1. In Situ Measurements

**Figure 6:**
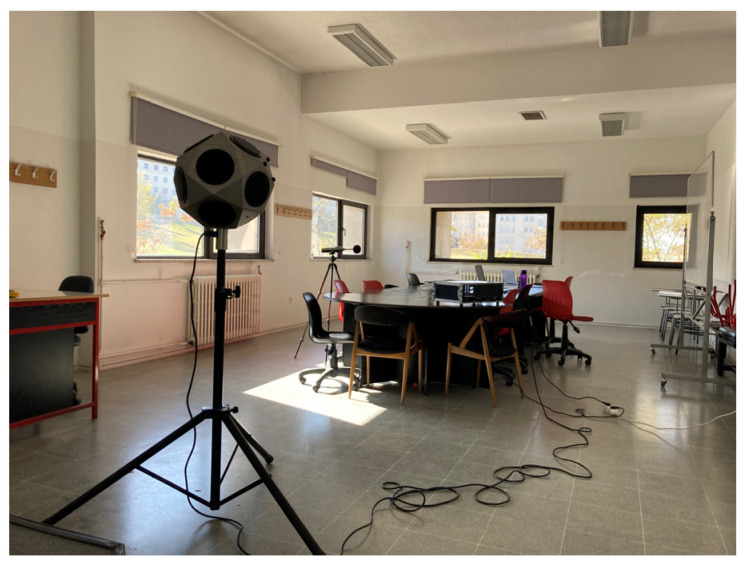
In situ measurements of the classroom.

The dimensions of the chosen classroom are 10.12 m long and 5.47 m wide. The height of the space was 3.6 m with a suspended ceiling of 0.5 m, as shown in Figure 6. The four-window classroom has the layout of a meeting room, since it is used only by graduate students, whose number is not more than twelve. To estimate the efficacity of the proposed materials when simulated, the classroom is field-tested in terms of its existing acoustic parameters. In situ measurements related to Reverberation Time (RT30,s), Equivalent Continuous A-Weighted Sound Level (L_Aeq,_ dBA), and Speech Transmission Index (STI) were conducted during weekends in unoccupied conditions, and the room was fully furnished with desks and chairs. 

**Figure 7:**
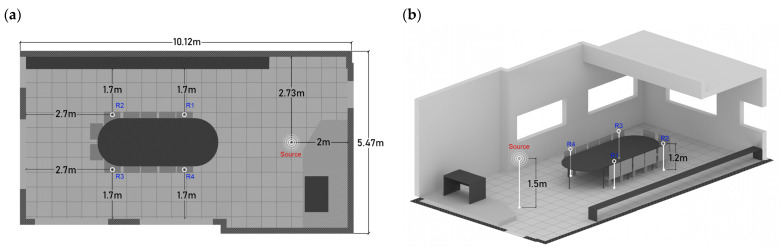
(**a**) Position of source and receivers; (**b**) Height of source and receivers.

The setup is formed by one source placed 2 m from the whiteboard, central with respect to the classroom’s layout, and four receivers having at least 1.5 m distance from the walls, as shown in Figure 7a. The height of the source (see Figure 7b) was set to 1.5 m from the ground (representing a standing teacher), whilst the receivers’ height was set to 1.2 m (representing sitting students). 

RT30 was measured in compliance with ISO 3382-2:2008—Acoustics—Measurement of room acoustic parameters—Part 2: Reverberation time in Ordinary Room standard [55] via the building acoustic analyser software DIRAC version 4.0. An e-sweep signal sampled at 48 kHz was emitted by a Bruel & Kjaer type 4006 omnidirectional speaker, acting as the source, supported by a B&K Power amplifier type 2716. The signal was recorded for each receiver position by a calibrated B&K 2230 Sound Level meter microphone. The extrapolation of data for T30 and STI was achieved using the same building acoustic analyser software. On the other hand, background noise levels, assessed in terms of L_Aeq,_ were measured in the receivers’ exact positions in the classroom using the B&K 2230 Sound Level meter. The average value of 40.1 dBA is less than the maximum accepted noise level (45 dBA) of classrooms in Turkey in unoccupied conditions [20].

#### 2.3.2. Numerical Simulations and Calibration of Models

**Figure 8:**
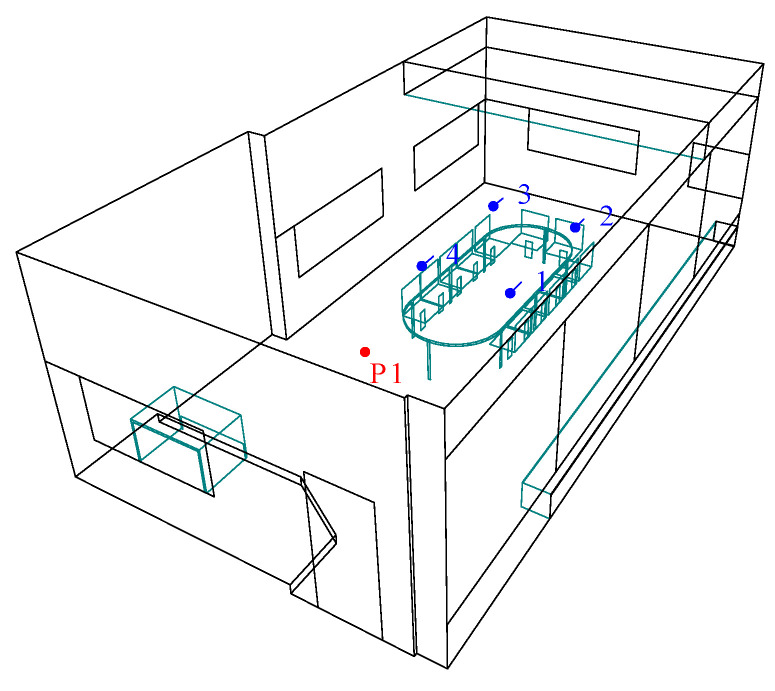
Source and receivers’ positions in ODEON simulations. Red color symbolizes the point source and blue color represents the receivers’ position in the room.

The 3D model of the classroom chosen as a case study was modelled using SketchUp and then imported into ODEON Acoustics Software version 16 (Education version) via the SU2Odeon plug-in. The modelling process was carried out according to the state-of-the-art recommendations for geometrical acoustics [56] by having surfaces larger than 0.35 m. The positions of the source and receivers remained the same (Figure 8), as explained in Section 2.3.1.

Sound absorption coefficients of materials were assigned based on previous literature [24,56] and adjusted accordingly through an iterative trial and error calibration process to achieve a good fit between the measured and simulated RT30. The calibration process was finalized when the difference between measured and simulated values of RT30 for each receiver was less than 5% of the measured value or Just-Noticeable Difference (JND) [55,57]. The final values of the modified absorption coefficients of the room materials are seen in Table 3. The scattering coefficient was set to 0.01 for all surfaces.

The classroom was acoustically assessed, modelled, and calibrated accordingly so that any treatment in the simulated model would also reflect the same behaviour in real-life acoustic conditions. Following this, one of the previously proposed prototypes showing the highest absorption coefficient through a wider frequency band was applied as a wall panel to the case study modelled in ODEON 16. The comparison between the classroom’s acoustic indexes regarding the before and after conditions yielded the effect this intervention had on the overall acoustic conditions of the space.

## 3. Results 

### 3.1. Impedance Tube Results

This section shows the theoretical and impedance tube results for the proposed MPP samples in Section 2.1.2 (Prototype 1) and Section 2.1.3 (Prototype 2). Employing the transfer function between the microphones, frequency-dependent measurements have been taken, and frequency–sound absorption coefficient graphics have been formed for the two cases.

- Prototype 1—One-layer parallel-arranged MPPs

**Figure 9:**
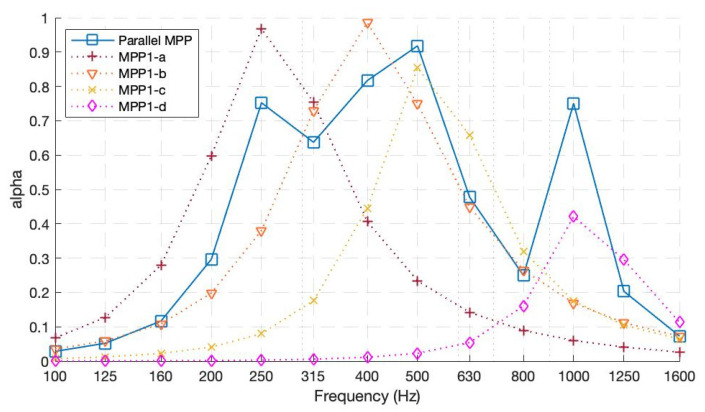
Theoretical absorption coefficients for Prototype 1 and each of its constituting MPPs.

The first prototype was composed of four parallel-arranged MPPS, each with different perforations and cavity lengths. Figure 9 shows the theoretical results of each constituting MPP and its overall performance when arranged in parallel. As inferred from Figure 9, each MPP has its highest absorption coefficient value at its resonant frequency, tuned accordingly by intuitive insights. When calculated separately, MPP1-a, having the lowest perforation ratio and largest cavity length, achieves an *α* value of 0.96 at 250 Hz. MPP1-b, which has a smaller-diameter hole and a back cavity of 60 mm, theoretically displays its resonant effect at 400 Hz with an *α* of 0.98. Moreover, MPP1-c has a maximum *α* value of 0.85 at 500 Hz. MPP1-d, having a hole diameter of 2 mm and the smallest back cavity (20 mm), aimed to target higher frequencies, yielding a maximum *α* value of 0.42 at 1000 Hz. Moreover, when the aforementioned MPPs are arranged in parallel, their absorption coefficient graph preserves each of the MPPs’ peaks and valleys, prevailing in an acceptable agreement with the theoretical model. Since each MPP was targeting different frequency bandwidths, their consequent peaks do not allow the parallel MPP absorption coefficient line curve to drop by preserving a good absorption on a broader frequency band.

**Figure 10:**
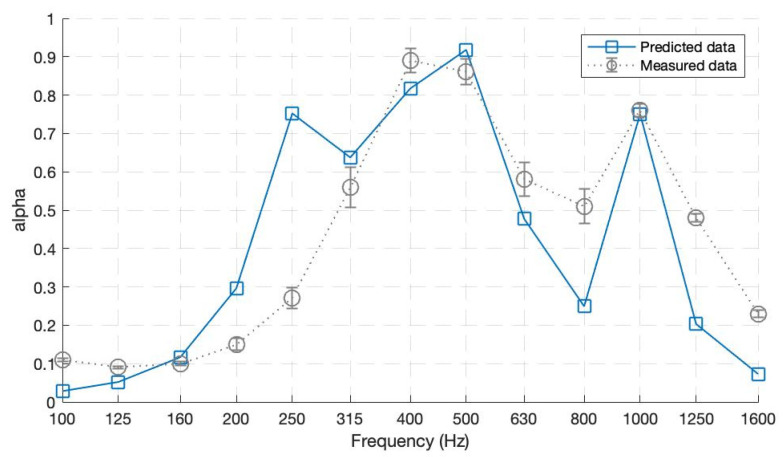
Absorption coefficients for Prototype 1—One layer with four parallel-arranged MPPs.

Figure 10, comparing the results of theoretically predicted and measured data from the impedance tube, shows a fair agreement between them. The predicted and measured data plots both follow the same trend of an *α* value of more than 0.5 from 300 to 1250 Hz. The measurements were conducted for three identical samples in a row. The low spread of error bars presented in Figure 10 shows the reproducibility of the materials. However, some discrepancies between the two lines are visible. The measurements report submitted by the Turkish Standards Institute states that the impedance tube method reliability decreases for low frequencies under 250 Hz, explaining the mismatch of the first peak in the predicted data plot. The very high absorption (*α* = 0.89 and 0.86) at 400 Hz and 500 Hz occurs due to the different resonances of MPP1-b and MPP1-c, respectively. The third peak of *α* = 0.79 at 1000 Hz is due to MPP1-d having a background cavity of only 20 mm and larger perforation holes, as explained previously. The parallel-arranged structure adds the resonances of the specific panels constituting at a larger absorption bandwidth, especially for the low–mid frequencies. Furthermore, some of the mismatches of dips and peaks may be because of the leakage of sound energy due to the non-enclosure of the material structure when inserted inside the impedance tube. Those fluctuations might have happened due to the transmission of sound waves from the frame’s joints or the latter’s possible vibrations. Furthermore, another critical point discussed by the authors of [37] is that there might be an effect of the impedance discontinuity on the MPP surfaces, affecting in this way the MPP results. Since the discontinuity effect was neglected during the theoretical predictions, it might have impacted the data plots obtained through experimental measures.

- Prototype 2—Double-layer parallel-arranged MPPs

**Figure 11:**
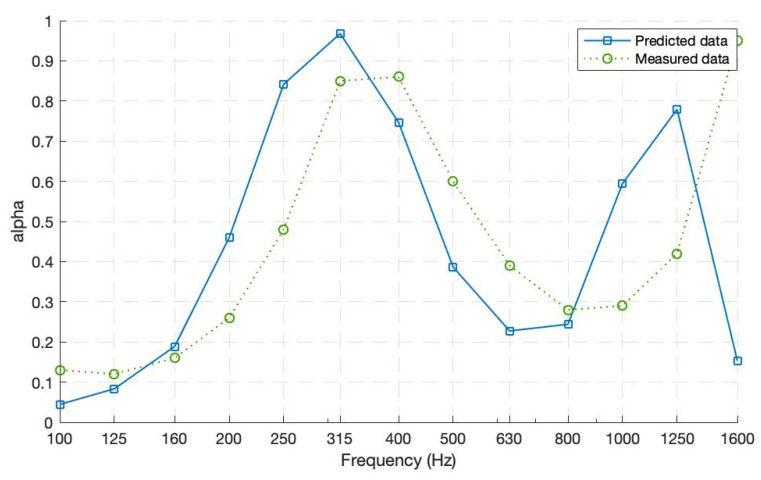
Absorption coefficients for Prototype 2—Double layer with four parallel-arranged MPPs each.

Even for the second case, when a more complex structure of MPPs was assessed, there is a fair agreement between the predicted and measured absorption curves (see Figure 11). The two absorption coefficient curves, including the peaks and valleys, remain consistent. The disparities between them might be because of the absence of the enveloping structure, potential vibrations of the partitions behind MPP1, and surface discontinuity between MPPs. This double-layer proposed structure of parallel-arranged MPPs has a wider absorption bandwidth, especially in the low frequencies (*α* ≤ 0.5 starting from 250 to 580 Hz). This occurs due to the different perforations at the first layer welcoming the incident wave. The back cavities (*D*2 = [80 mm, 60 mm, 40 mm, 20 mm]) after the second layer are where most of the resonance effects occur. Another resonance peak is present at a higher-frequency range due to the small cavity (*D*1 = 20 mm) between the two layers. 

Since Prototype 1 showed a more significant absorption coefficient for a wider frequency band, which corresponds with the speech frequency range, it is evaluated for its performance at a room acoustic scale, as explained in the following sections.

### 3.2. Geometrical Acoustics Results

**Figure 12:**
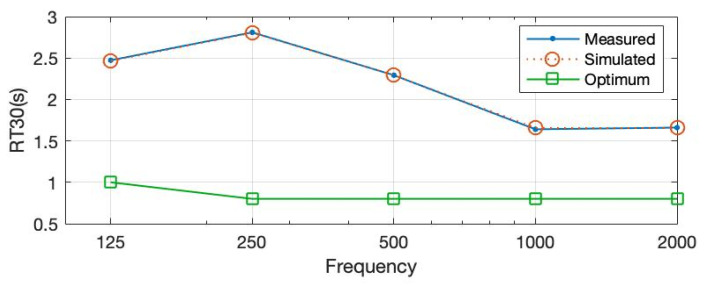
RT30 values for measured and simulated existing conditions compared to optimum ones.

The field-measured and calibrated RT30 values of the case study explained in Section 2.3 are presented in Figure 12. The existing values do not comply with the ones proposed by Standards [16,17,18,19,20] referring to corresponding building typology and volume. In classrooms smaller than 250 m^3^, the RT30 should not exceed 0.8 s at mid frequencies (500–1000 Hz) for furnished spaces in unoccupied conditions. In the assessed case study, those values lie far above the recommended thresholds, averaging 2 s in mid frequencies. 

On the other hand, ODEON, a geometrical acoustics software, requires a random incidence absorption coefficient of newly introduced materials that can be obtained from purely energetic measurement methods. In such programs, sound propagation in the space is formulated by ray tracing and image source approaches. It configures sound rays traveling in different directions in the room, resembling their behaviour in real-life acoustic scenarios but neglecting their wave-like nature. As the parallel-arranged MPP in Prototype 1 provides the impedance and absorption coefficient data collected from normal incidence, the conversion to random absorption coefficient was needed. The literature agrees on approximating the random incidence value by only measuring the normal incidence when the surface is locally reacting, as in this study’s case. Theoretically, a single calculation at *θ* = 50° for Equation (13) might be sufficient, being the same as the random incidence value [24]. The absorption coefficients are then calculated by Equation (13). The assigned random incidence absorption values for the proposed one-layer parallel-arranged MPPs are listed in Table 4.

(13) $$\alpha =1-{\left|\frac{(Z_{tot}\cos\theta )-\rho c}{(Z_{tot}\cos\theta )+\rho c}\right|}^{2}$$

**Figure 13:**
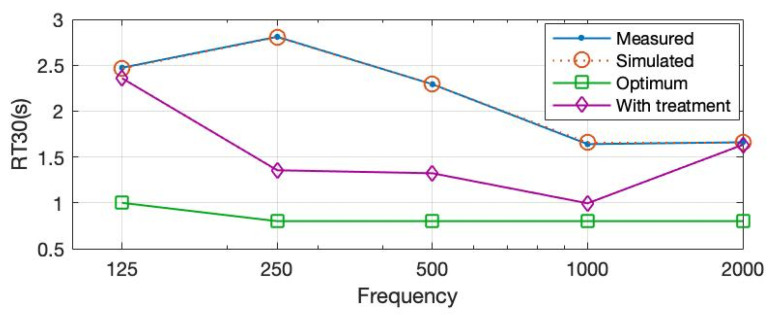
RT30 values after the proposed material is used as treatment compared to optimum and existing values.

Prototype 1 is introduced and applied to the simulated and calibrated ODEON model in the form of a wall treatment covering the side wall with no openings. Nine panels having 1200 × 2400 mm sizes are mounted in a steel stud wall frame with a surface of nearly 25 m^2^. After the classroom is acoustically treated in this way, the RT30 values reduce significantly. As seen in Figure 13, the RT30 values decrease by more than 1 s for 250 Hz and 500 Hz frequencies corresponding to 1.3 s each. Furthermore, a significant reduction in the reverberant sound happens at 1000 Hz, resulting in an RT30 of 0.9 s. Even though it is still outside of the range of optimized values, this acoustical treatment shows a significant improvement in the space without any volume change.

Apart from RT30, acoustic comfort in terms of speech intelligibility in classrooms was also objectively assessed by Speech Transmission Index (STI) values. As a speech metric, the STI evaluates the impact of noise and room reflections on speech intelligibility from the source to the receivers. Its values, with their corresponding evaluations, are shown in Table 5.

For this case study, assumptions for STI are taken in compliance with Noise Criteria 45 (NC45). For the four receivers in the room shown in Table 6, STI values lie in the Fair range [58], starting from 0.48 for R1 and R3 and reaching 0.5 for R2. The value for R4 is 0.49. When the same classroom is treated with Prototype 1 as a wall panel instead of plaster, the STI values for each receiver improve to 0.55. While the former values faced the limits of the “Poor” range, the latter tended towards “Good” speech intelligibility. This improvement is significant for spaces whose primary purpose is speech, such as classrooms.

## 4. Conclusions

The current study investigated different arrangements of transparent MPP absorbers using the Equivalent Circuit Model (ECM). These structures can be an alternative to conventional absorbing materials in architectural applications to attenuate a broader range of frequencies. Many studies [47,49,50,59] have investigated different combinations to address the narrow bandwidth of MPPs, but to the authors’ knowledge, none have continued to assess the performance of those materials in architectural applications. 

This work proposed two prototypes with different MPP arrangements. They were numerically modelled, manufactured, and evaluated for their normal incidence sound absorption coefficients in an impedance tube. The agreement between the theoretical predictions by the ECM model and the experimental data obtained by the impedance tube was at an acceptable level for both prototypes. Prototype 1, consisting of one layer composed of four different MPPs facing different cavities, showed a wider absorption bandwidth in the low–mid frequency spectrum (*α* ≥ 0.5 from 300 to 1250 Hz). Despite some discrepancies, which literature has shown to be quite common in those complex structures [49,50,59], the results indicate a broadening of the absorption coefficient curve because of the multiple resonance peaks each panel introduces. Prototype 2 was composed of two layers having different parallel-arranged MPPs cascaded one after the other, with a distance (*D*1) in between and various partitioned cavities (*D*2) after the second layer. Its results show that absorption at low frequencies is related to the different perforation ratios of MPP1 and the lengths of *D*2. Moreover, the second peak in the higher-frequency range is associated with the short length of *D*1. Because Prototype 1 showed a better performance in reducing sound amplitude for a wide band, it was introduced to a classroom chosen as a case study. The RT30 values, averaging 2 s for mid frequencies in the existing conditions, decreased to an average of 1.1 s for frequencies ranging from 250 to 1000 Hz after the material was applied as a treatment. The same improvement trend was observed for the STI values of the receivers. 

This pilot work showed preliminary insights into how the parameters could be tuned to handle specific target frequencies. Still, the decision on the 4 n parameters of the proposed structures was based on trial and error procedures, which did not provide the best absorption curve possible. For further studies, it would be beneficial to rely on optimization algorithms, as recent studies have benefited from [59,60] by strongly considering the constraints of existing building and finishing materials. Another interesting trail to follow in further work from the design perspective might be surface design in terms of visual patterns. This approach would behave as a trade-off between visual aesthetics and functional acoustics, which studies have attempted to achieve lately [61,62,63,64]. 

Although the design of the arrangements proposed in this study may be relatively complex and might face application difficulties, the presented approach might be an alternative solution in spaces where the thickness of the panels and the usage of fibrous/porous materials is a critical constraint. As the second part of this study addressed, indoor education environments have significant problems related to amplified noise and speech intelligibility [5,6,7]. The literature emphasizes that prolonged exposure to excessive levels of noise can lead to several adverse human health issues such as fatigue, stress, hearing loss, high blood pressure, sleeping disorders, psychological disorders, hypertension, obesity, cognitive impairment in children, coronary heart disease, and diabetes type I and II [65,66,67]. Along this line, the proposed materials pose great potential in interior architecture as fibreless transparent absorbers that can cope with the highest hygiene demands by having a tuneable design according to the needs of the space. Investigations such as this work from the architect’s point of view help the latter make informed decisions for incorporating better sound within a project’s design, ultimately delivering a better user experience.

## Figures and Tables

**Table 1:** Parameters of four parallel-arranged MPPs.

	MPP1			
	*d* (mm)	*t* (mm)	*p* (%)	*D* (mm)
MPP1-a	1	6	1.6	80
MPP1-b	0.7	6	2.8	60
MPP1-c	1	6	3.14	40
MPP1-d	2	6	6.4	20

**Table 2:** Parameters of the two layers each having four parallel-arranged MPPs.

	MPP1					MPP2			
	*d* (mm)	*t* (mm)	*p* (%)	*D*1 (mm)		*d* (mm)	*t* (mm)	*p* (%)	*D*2 (mm)
MPP1-a	1	6	4.9	20	MPP2-a	1	6	1.6	60
MPP1-b	1	6	1.6	20	MPP2-b	0.7	6	2.8	20
MPP1-c	2	6	6.4	20	MPP2-c	1	6	4.9	80
MPP1-d	0.7	6	2.8	20	MPP2-d	2	6	6.4	40

**Table 3:** Modified absorption coefficients of materials in the room over 1/1 octave bands.

Surfaces	Materials	125 Hz	250 Hz	500 Hz	1000 Hz	2000 Hz
Floor	Ceramic tile	0.01	0.01	0.02	0.02	0.02
	Thin carpet on underlay	0.03	0.09	0.3	0.54	0.5
Wall	Plaster on concrete	0.03	0.03	0.02	0.03	0.04
Ceiling	Plaster on concrete	0.03	0.03	0.02	0.03	0.04
Windows	Ordinary window glass	0.35	0.25	0.18	0.12	0.07
Door	Wooden door	0.14	0.1	0.06	0.08	0.1
Furniture	Wooden desks	0.14	0.07	0.21	0.25	0.15
	Plastic/wooden chairs	0.06	0.1	0.2	0.4	0.35

**Table 4:** Random absorption coefficients for the proposed one-layer parallel-arranged MPPs.

Prototype 1	125 Hz	250 Hz	500 Hz	1000 Hz	2000 Hz
One-layer parallel-arranged MPPs with different cavities	0.05	0.76	0.92	0.75	0.04

**Table 5:** STI values and their corresponding evaluation.

STI ranges	0.00–0.30	0.30–0.45	0.45–60	0.60–0.75	0.75–1.00
Evaluation	bad	data	fair	good	excellent

**Table 6:** STI values for each receiver before and after treatment.

STI	R1	R2	R3	R4
Before MPP treatment	0.48	0.5	0.48	0.49
After MPP treatment	0.55	0.55	0.55	0.55

## Data Availability

The data presented in this study are available on request from the corresponding author.
